# Effect of epicardial fat volume on outcomes after left atrial posterior wall isolation in addition to pulmonary vein isolation in patients with persistent atrial fibrillation

**DOI:** 10.3389/fcvm.2022.1005760

**Published:** 2022-10-26

**Authors:** Daehoon Kim, Hee Tae Yu, Oh-Seok Kwon, Tae-Hoon Kim, Jae-Sun Uhm, Boyoung Joung, Moon-Hyoung Lee, Hui-Nam Pak

**Affiliations:** Division of Cardiology, Department of Internal Medicine, Yonsei University Health System, Seoul, Republic of Korea

**Keywords:** atrial fibrillation, epicardial adipose tissue, posterior wall isolation, catheter ablation—atrial fibrillation, rhythm outcome

## Abstract

**Background:**

Greater epicardial adipose tissue (EAT) is related to higher recurrences after atrial fibrillation catheter ablation (AFCA). We investigated the effects of posterior wall box isolation (POBI) in conjunction with circumferential pulmonary vein isolation (CPVI) on rhythm outcomes according to varying EAT volumes among patients with persistent atrial fibrillation (PeAF).

**Materials and methods:**

We included 1,187 patients with PeAF undergoing a *de novo* AFCA including those receiving CPVI alone (*n* = 687) and those receiving additional POBI (*n* = 500). The rhythm outcomes at 2 years post-AFCA were compared in subgroups stratified by the EAT volume using propensity overlap weighting.

**Results:**

A reduced EAT volume was linearly associated with more favorable rhythm outcomes for additional POBI than for CPVI alone (P for interaction = 0.002). Among the patients with smaller EAT volumes (≤116.23 mL, the median value, *n* = 594), additional POBI was associated with a reduced AF recurrence risk as compared to CPVI only [weighted HR (hazard ratio) 0.74, 95% CI (confidence interval) 0.56–0.99]. In contrast, among the remaining 593 patients with greater EAT volumes (>116.23 mL), No difference was observed in the recurrence risk between the additional POBI and CPVI alone groups (weighted HR 1.13, 95% CI 0.84–1.52). Among 205 patients with repeat ablations, the POBI reconnection rate was more frequent in the large EAT group (77.4%) than in the small EAT group (56.7%, *P* = 0.034).

**Conclusion:**

While PeAF patients with a smaller EAT volume averted AF recurrence by additional POBI after CPVI, no benefit of the POBI was observed in those with a greater EAT volume.

## Introduction

Atrial fibrillation (AF), the most common sustained cardiac arrhythmia, increases the risk of morbidity and mortality arising owing to strokes and congestive heart failure. Those events are associated with a high burden of healthcare costs (1). Obesity is regarded as a modifiable risk factor for AF (2), and the mechanism of its occurrence is unclear; however, it might be mediated by epicardial adipose tissue (EAT) (3, 4). A strong relationship between the body mass index (BMI) and amount of EAT has been suggested (5). Abnormal EAT is a source of proinflammatory and fibrotic molecules that can affect the adjacent atria (6). Several studies have revealed a relationship between increased EAT and the AF severity and remodeling of the left atrium (LA) (3, 7). Increased EAT is also related to a higher risk of clinical recurrence after AF catheter ablation (AFCA) (3, 8).

Catheter ablation is an effective treatment for AF as it reduces the number of acute episodes and prolongs the maintenance of sinus rhythm. Circumferential pulmonary vein (PV) isolation (CPVI) is the cornerstone technique of AFCA. However, extra-PV triggers play an important role in pathophysiology of persistent AF (PeAF), and a CPVI alone generally does not achieve a satisfactory clinical outcome. The creation of linear lesions in the LA in addition to a CPVI has been widely adopted as the strategy of substrate modification for AF (9). While electrical isolation of the LA posterior wall [posterior box isolation (POBI)] has been reported to be beneficial (10), it does not improve the rhythm outcomes as compared to a CPVI alone in patients with PeAF from a randomized clinical trial (RCT) by Lee et al. (11). Identifying patients who may benefit from additional linear ablation remains an important clinical question. We hypothesized that the effect of a POBI added to the CPVI, as compared to a CPVI alone, varies per the EAT volume in patients with AF.

## Materials and methods

### Study population

**FIGURE 1:**
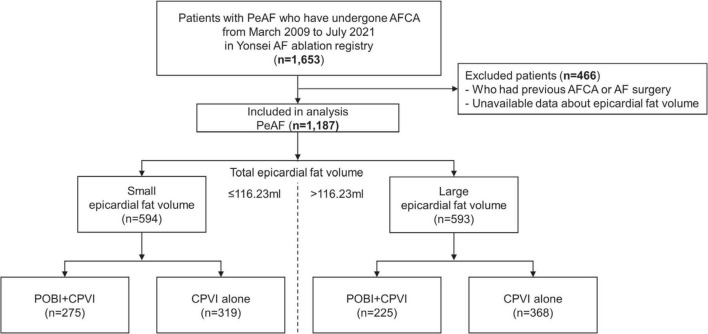
Flow chart for the present study. AF, atrial fibrillation; AFCA, atrial fibrillation catheter ablation; CPVI, circumferential pulmonary vein isolation; EAT, epicardial adipose tissue; PeAF, persistent atrial fibrillation; POBI, posterior wall box isolation.

This study was a single-center retrospective observational cohort study. The study protocol adhered to the Declaration of Helsinki and was approved by the Yonsei University Health System’s institutional review board. We obtained written informed consent from all patients included in the Yonsei AF Ablation Cohort Database (NCT02138695). Among the 1,653 patients who received AFCA for symptomatic drug-refractory PeAF between March 2009 and July 2021 at a tertiary referral center (Yonsei University Severance Hospital, Republic of Korea), 1,187 patients with available data regarding their EAT volume at the time of the ablation procedure and without a previous AFCA or AF surgery were enrolled in this study (Figure 1). All antiarrhythmic drugs (AADs) were discontinued for at least five half-lives, and amiodarone was discontinued for more than a month prior to the procedure.

### Measurement of epicardial adipose tissue

**FIGURE 2:**
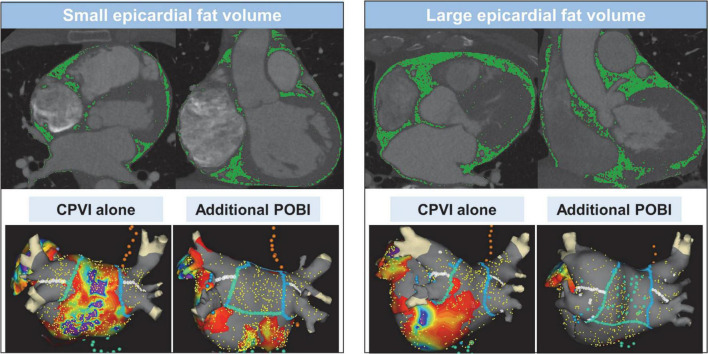
Measurement of epicardial adipose tissue volume using 3-dimensional computed tomography and ablation lesions and voltage map of the strategies of circumferential pulmonary vein isolation alone and additional posterior box isolation in patients with small or large epicardial adipose tissue volume. CPVI, circumferential pulmonary vein isolation; POBI, posterior wall box isolation.

Computed tomography (CT) images of all patients were obtained *via* the electrocardiography (ECG)-gated method before the AFCA (Figure 2). EAT was defined as the adipose tissue located between the myocardium and pericardium. The CT attenuation threshold for detection of the adipose tissue ranged from −190 to −30 Hounsfield units, as used in previous studies (4). The procedure for measuring the EAT was detailed in a previous study that utilized 600 patients (8). Briefly, the pericardial contours were obtained using a three-dimensional active tool after placing 10–15 control points on the pericardium at intervals of 10 mm from the superior border of the pulmonary artery bifurcation to the most distal end of the left ventricle. Subsequently, a CT attenuation threshold was applied inside the pericardial contour to obtain adipose tissue and, if deemed appropriate, fine-tuned using the paintbrush tool. The EAT volume was calculated using the final obtained fat voxels. Then, the EATs of the atrium and the ventricles were separated based on the tricuspid and mitral valve, as shown in Supplementary Figure 1. This procedure to quantify the EAT was performed using an application software [ITK-SNAP; Penn Image Computing, Science Laboratory (PICSL), University of Pennsylvania, PA, USA, and AMBER; Laonmed Inc., Seoul, Korea] by two independent investigators. The intraobserver and interobserver reproducibility of EAT analysis was assessed in 10 randomly selected samples using intraclass correlation coefficient (ICC) analysis.

### Electrophysiological mapping and radiofrequency catheter ablation

The details of the electrophysiological mapping and AFCA technique are presented in Supplementary Methods. Figure 2 depicts the examples of ablation lesions and voltage map after catheter ablation in the CPVI alone and POBI groups. All patients received a CPVI during the procedure. Of the enrolled patients, 42.1% (500/1,187) received linear ablation along the roof and posterior-inferior wall by connecting both sides of the CPVI at the top and bottom levels, respectively. We defined a POBI as a voltage abatement of < 0.1 mV on the LA posterior wall with successful bidirectional block of the roof line. After a successful bidirectional block of the roof line, if the POBI could not be achieved after two attempts of a posterior inferior line ablation, we eliminated the remnant potential in the posterior LA by touch-up ablation. After completion of the protocol-based ablation, the procedure was ended when AF recurrence was not observed within 10 min after cardioversion with an isoproterenol infusion (5–20 μg/min depending on ß-blocker use, target sinus heart rate, 120 bpm).

### Post-ablation management, follow-up, and clinical outcome

The details of management and follow-up after AFCA are presented in Supplementary Methods. If recurrent AF or atrial tachycardia (AT) was observed repeatedly under AADs after the *de novo* AFCA, a repeat AFCA was recommended. The details about the repeat ablation procedures have been addressed previously (12).

The primary outcome was a clinical recurrence, defined as any episode of AF or AT lasting for at least 30 s occurring more than 3 months during 2 years of follow-up after the procedure. Any documented AF or AT within a 3-month blanking period was defined as early recurrence. An ECG was performed in all patients visiting the outpatient clinic at 1, 3, 6, and 12 months following the AFCA and every 6 months thereafter or whenever symptoms developed. Twenty-four-hour Holter recordings were performed at 3, 6, and 12 months and every 6 months thereafter. Patients who reported episodes of palpitations suggestive of an arrhythmia recurrence received Holter monitoring or event monitoring recordings.

### Statistical analysis

Continuous variables are presented as the mean ± standard deviation or median (interquartile range) and compared using the Student’s *t*-test or Mann-Whitney *U-*test, while categorical variables are presented as frequencies (percentages) and compared using either the chi-square test or Fisher’s exact test. To analyze the EAT-dependent effect of an additional POBI on the primary outcome, a multivariable Cox regression model was fitted to the entire study population using an interaction term for the total, atrial, or ventricular EAT volume (modeled as a cubic spline) and ablation strategies (additional POBI or CPVI alone). The age, sex, BMI, CHA_2_DS_2_-VASc score, medical history, and echocardiographic parameters were included as covariates in the regression models. Standard errors were computed using 1,000 bootstrap replicates.

Subgroup analyses stratified by the median total EAT volume (116.23 mL) were performed using propensity overlap weighting to account for the differences in the baseline characteristics between the patients who received an additional POBI and those who received a CPVI alone in each subgroup. The details of the propensity overlap weighting are presented in Supplementary Methods. The distribution of the propensity scores before and after propensity weighting is shown in Supplementary Figure 2. We compared the incidence of the outcomes using the weighted log-rank test and plotted the weighted Kaplan-Meier survival curves. We used a Cox regression to estimate the relative hazards of primary outcome. Cofactors that were not balanced by weighting were included as covariates in the Cox regression analysis. The statistical analyses were performed using R version 4.1.1 software (The R Foundation).^1^

## Results

Table 1 presents the baseline characteristics of the patients with PeAF who underwent catheter ablation stratified according to the median value of the total EAT volume. The median age was 60 (interquartile range 53–67) years, and 20.4% were female. Patients with a greater EAT volume were likely to be older and had a higher BMI, higher CHA_2_DS_2_-VASc score, higher prevalence of hypertension and diabetes, larger LA, and higher E/Em than those with a smaller volume. Intra and inter-observer ICC in the measurement of EAT were 0.996 and 0.994, respectively.

**TABLE 1:** Baseline clinical characteristics.

Variables	Overall	Small EAT (≤116.23 mL)	Large EAT (>116.23 mL)	*P*-value
	(*n* = 1,187)	(*n* = 594)	(*n* = 593)	
Age, years	60(53−67)	59(52−66)	60(53−68)	0.006
Female	242 (20.4)	156 (26.3)	86 (14.5)	< 0.001
BMI, kg/m^2^	25.3(23.5−27.4)	24.2(22.6−25.9)	26.6(24.6−28.4)	< 0.001
CHA_2_DS_2_-VASc score	2(1−3)	1(1−2)	2(1−3)	0.001
Comorbidities				
Heart failure	249 (21.0)	122 (20.5)	127 (21.4)	0.764
Hypertension	610 (51.4)	256 (43.1)	354 (59.7)	< 0.001
Diabetes mellitus	230 (19.4)	83 (14.0)	147 (24.8)	< 0.001
Stroke	123 (10.4)	63 (10.6)	60 (10.1)	0.857
Vascular disease	119 (10.0)	49 (8.2)	70 (11.8)	0.052
Echocardiographic parameters				
LA dimension, mm	44(40−48)	43(39−47)	45(42−49)	< 0.001
LVEF,%	62(57−67)	62(57−67)	62(57−66)	0.310
E/Em	9.3(7.6−12.1)	9.0(7.2−11.8)	9.9(8.0−12.7)	< 0.001
AAD use before the ablation				
Class Ic	547 (46.1)	282 (47.5)	265 (44.7)	0.366
Class III	751 (63.3)	365 (61.4)	386 (65.1)	0.214
Total EAT volume, mL	116.2(87.1−149.2)	87.1(69.9−102.1)	149.2(132.5−172.8)	< 0.001
Atrial EAT volume, mL	48.2(35.5−63.0)	35.6(27.6−42.7)	63.0(53.8−73.9)	< 0.001
Ventricular EAT volume, mL	67.5(50.7−86.0)	50.7(39.8−59.3)	86.0(75.1−103.3)	< 0.001
Mean LA wall thickness, mm	1.91(1.67−2.13)	1.93(1.72−2.14)	1.89(1.63−2.09)	0.007
Mean LA voltage, mV	1.06(0.64−1.59)	1.03(0.61−1.58)	1.10(0.67−1.61)	0.150

Values are presented as the median (interquartile range) or number (%). AAD, antiarrhythmic drug; AF, atrial fibrillation; BMI, body mass index; EAT, epicardial adipose tissue; E/Em, mitral inflow velocity/mitral annulus tissue velocity; LA, left atrium; LVEF, left ventricular ejection fraction.

### Epicardial adipose tissue volume and the relative benefit from posterior wall box isolation

**FIGURE 3:**
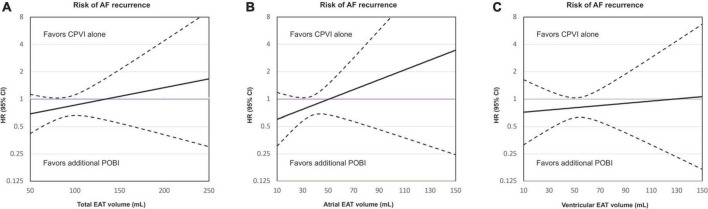
Relationship between the total epicardial adipose tissue (EAT) volume **(A)**, atrial EAT volume **(B)**, and ventricular EAT volume **(C)** and risk of a clinical recurrence in the group receiving only a circumferential pulmonary vein isolation (CPVI) and the group receiving an additional posterior wall box isolation (POBI). The *x*-axis shows the total EAT volume and the *y*-axis shows the hazard ratios (HRs) for clinical recurrence associated with the additional POBI treatment as compared to a CPVI treatment alone. The purple horizontal line indicates HR = 1, which corresponds to an equal risk of outcomes in patients treated with an additional POBI treatment as compared to those receiving a CPVI alone. The dashed black lines indicate the 95% confidence intervals (CIs). AF, atrial fibrillation; CPVI, circumferential pulmonary vein isolation; EAT, epicardial adipose tissue; HR, hazard ratio; POBI, posterior wall box isolation.

Figure 3A shows a comparison between the strategies of a CPVI alone and an additional POBI in terms of the relationship between the total EAT volume and risk of a clinical recurrence outcome. The treatment benefits of an additional POBI varied as a function of the total EAT volume. With a smaller EAT volume, a linearly increasing association was observed between an additional POBI and a lower risk of clinical recurrence when compared with that of a CPVI alone. The beneficial effects of an additional POBI on the rhythm outcome compared to a CPVI alone were more prominent with a smaller atrial EAT volume (Figure 3B) than with a smaller ventricular EAT volume (Figure 3C).

Subgroup analyses in which the patients were stratified according to the median value of the total EAT volume in the patients included in this study (116.23 mL) showed that there was an interaction between the EAT volume and the effect of the additional POBI on clinical recurrence (P for interaction = 0.002).

### Role of posterior wall box isolation in patients with a smaller epicardial adipose tissue volume

Among 594 patients with a total EAT volume of ≤ 116.23 mL (the median value in this cohort), 319 and 275 patients underwent a CPVI alone and an additional POBI, respectively. The median follow-up duration was 20 months. Patients in the additional POBI group more frequently had a history of vascular disease and had a larger LA size than those in the CPVI alone group (Table 2). After overlap weighting, all the characteristics were balanced between the groups (Supplementary Table 1). The ablation time (median: 6,140 vs. 2,665 s) was longer in the additional POBI group than in the CPVI alone group (Table 2). There was no difference in the major complication rates between the two groups (2.2% in the additional POBI group vs. 2.2% in the CPVI alone group).

**TABLE 2:** Clinical and procedure-related characteristics.

	Small EAT (≤116.23 mL) (*n* = 594)			Large EAT (>116.23 mL) (*n* = 593)		
	CPVI alone (*n* = 319)	Additional POBI (*n* = 275)	*P*-value	CPVI alone (*n* = 368)	Additional POBI (*n* = 225)	*P*-value
Age, years	59 (52–66)	59 (53–66)	0.993	60 (53–67.25)	60 (53–68)	0.890
Female	81 (25.4)	75 (27.3)	0.670	54 (14.7)	32 (14.2)	0.975
BMI, kg/m^2^	24.2 (22.5–26.0)	24.0 (22.6–25.8)	0.785	26.5 (24.5–28.4)	26.7 (24.8–28.5)	0.533
CHA_2_DS_2_-VASc score	2 (1–2)	1 (1–3)	0.644	2 (1–3)	2 (1–3)	0.849
Comorbidities						
Heart failure	68 (21.3)	54 (19.6)	0.687	86 (23.4)	41 (18.2)	0.168
Hypertension	137 (42.9)	119 (43.3)	1.000	211 (57.3)	143 (63.6)	0.158
Diabetes mellitus	47 (14.7)	36 (13.1)	0.648	101 (27.4)	46 (20.4)	0.069
Stroke	33 (10.3)	30 (10.9)	0.929	33 (9.0)	27 (12.0)	0.295
Vascular disease	18 (5.6)	31 (11.3)	0.019	35 (9.5)	35 (15.6)	0.037
Echocardiographic parameters						
LA dimension, mm	42 (38–46)	44 (40–48)	< 0.001	44 (41–48)	48 (44–51)	< 0.001
LVEF,%	62 (57–67)	62 (58–67)	0.801	62 (58–65.62)	62 (57–67)	0.962
E/Em	9.0 (7.2–10.8)	9.2 (7.2–12.1)	0.019	9.53 (8–12.17)	10 (8–13.29)	0.038
AAD use before the ablation						
Class Ic	141 (44.2)	141 (51.3)	< 0.001	161 (43.8)	104 (46.2)	0.615
Class III	205 (64.3)	160 (58.2)	0.152	240 (65.2)	146 (64.9)	1.000
AAD use after the ablation						
At discharge	140 (43.9)	102 (37.1)	0.110	169 (45.9)	90 (40.0)	0.185
After 3 months	159 (49.8)	139 (50.5)	0.930	186 (50.5)	106 (47.1)	0.467
Total EAT volume, mL	89.3 (70.3–104.3)	84.9 (68.3–100.4)	0.031	150.1 (132.5–170.7)	148.4 (132.5–178.8)	0.333
Atrial EAT volume, mL	36.7 (29.6–43.9)	34.3 (26.6–41.1)	0.006	63.6 (55.0–73.6)	61.9 (52.4–75.2)	0.190
Ventricular EAT volume, mL	50.9 (40.7–59.5)	50.1 (39.1–58.9)	0.340	85.4 (75.1–100.6)	88.0 (75.2–105.7)	0.438
Procedure-related characteristics						
Ablation time, sec	2,665 (1,683–4,226)	6,140 (5,145–7,018)	< 0.001	2,392 (1,575–3,536)	6,270 (4,638–7,298)	< 0.001
Complications	14 (4.4)	15 (5.5)	1.000	10 (2.7)	7 (3.1)	0.980
Major complications	7 (2.2)	6 (2.2)	1.000	7 (1.9)	3 (1.3)	0.847
Atrioesophageal fistula	0	1		0	0	
Cardiac tamponade	7	2		7	3	
Phrenic nerve palsy	1	1		0	0	

Values are presented as the median (interquartile range) or number (%). AAD, antiarrhythmic drug; BMI, body mass index; CPVI, circumferential pulmonary vein isolation; EAT, epicardial adipose tissue; E/Em, mitral inflow velocity/mitral annulus tissue velocity; LA, left atrium; LVEF, left ventricle ejection fraction; POBI, posterior wall box isolation.

**FIGURE 4:**
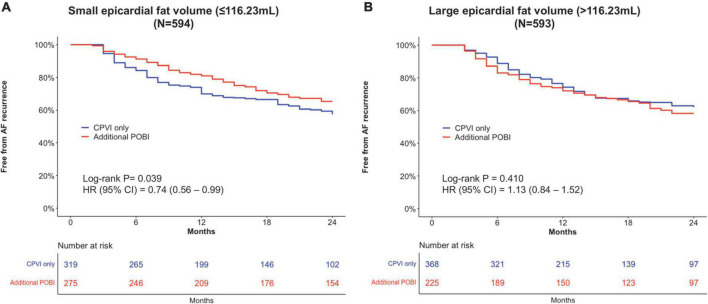
Kaplan–Meier analyses of the clinical recurrence-free survival in patients with a small total epicardial adipose tissue EAT volume **(A)** and in patients with a large EAT volume **(B)**. AF, atrial fibrillation; CI, confidence interval; CPVI, circumferential pulmonary vein isolation; HR, hazard ratio; POBI, posterior wall box isolation.

After overlap weighting, the early recurrence rate was not different between the CPVI alone and additional POBI groups (48.3% vs. 47.3%, *P* = 0.812) (Table 3). The cumulative incidence of clinical recurrence at 2 years after the AFCA was lower in the additional POBI group (34.7%) than CPVI alone group (42.5%) (weighted log-rank *P* = 0.039) (Figure 4A). Among those with early or clinical recurrence, the proportions of AT were higher in the additional POBI than in the CPVI alone group (*P* < 0.001 for early recurrence; *P* = 0.014 for clinical recurrence). Performing the POBI additionally was associated with a 26% decreased risk of clinical recurrence as compared to a CPVI alone [hazard ratio (HR) 0.74, 95% confidence interval (CI) 0.56–0.99].

**TABLE 3:** Rhythm outcomes.

	Small EAT (≤116.23 mL)			Large EAT (>116.23 mL)		
	CPVI alone (*n* = 319)	Additional POBI (*n* = 275)	*P*-value	CPVI alone (*n* = 368)	Additional POBI (*n* = 225)	*P*-value
**Early recurrence**						
Crude event number (%)	153 (48.0)	129 (46.9)	0.862	193 (52.4)	89 (39.6)	0.003
Recurrence type AF, *n* (% in early recurrence)	131 (85.6)	86 (66.7)	< 0.001	176 (91.2)	54 (60.7)	< 0.001
Recurrence type AT, *n* (% in early recurrence)	22 (14.4)	43 (33.3)		17 (8.8)	35 (39.3)	
Weighted%	48.3	47.3	0.812	54.1	39.4	0.001
**Clinical recurrence**						
Crude event number (%)	111 (34.8)	91 (33.1)	0.726	109 (29.6)	86 (38.2)	0.038
Recurrence type AF, *n* (% in recurrence)	95 (85.6)	64 (70.3)	0.014	100 (91.7)	57 (66.3)	< 0.001
Recurrence type AT, *n* (% in recurrence)	16 (14.4)	27 (29.7)		9 (8.3)	29 (33.7)	
Weighted%	36.6	32.5	0.313	30.8	38.5	0.070
Weighted hazard ratio (95% CI)	1 (ref)	0.74 (0.56–0.99)	0.040	1 (ref)	1.13 (0.84–1.52)	0.409

AF, atrial fibrillation; AT, atrial tachycardia; CI, confidence interval; CPVI, circumferential pulmonary vein isolation; EAT, epicardial adipose tissue; POBI, posterior wall box isolation.

### Additional posterior wall box isolation in patients with a larger epicardial adipose tissue volume

Among the 593 patients with a total EAT volume > the median value, 368 and 225 patients underwent a CPVI alone and an additional POBI, respectively. The median follow-up duration was 15 months. Similar to the observations in those with a small EAT volume, the patients undergoing an additional POBI had a larger LA size than those undergoing a CPVI alone (Table 2). After overlap weighting, all the characteristics were balanced between the groups (Supplementary Table 2). The ablation time was longer in the additional POBI group (median: 6,270 s) than in the CPVI alone group (median 2,392 s) (Table 2). The major complication rates did not differ between the two groups (1.3% in the additional POBI group vs. 1.9% in the CPVI alone group).

After overlap weighting, the early recurrence was less frequent in the additional POBI group (39.4%) than in the CPVI alone group (54.1%) (*P* = 0.001) (Table 3). The cumulative incidence of clinical recurrence did not differ between the groups (41.7% in the additional POBI group vs. 38.0% in the CPVI alone group, weighted log-rank *P* = 0.409; HR 1.13, 95% CI 0.84–1.52) (Figure 4B). Among those with early or clinical recurrence, the proportions of AT were higher in the additional POBI than in the CPVI alone group (*P* < 0.001 for both early and clinical recurrence).

### Findings of redo ablation

Repeat ablation was performed in 102 and 103 patients in the small and large EAT groups, respectively (Table 4). The time interval between the index procedure and repeat procedure did not differ according to the EAT volume (22 months in the small EAT vs. 20 months in the large EAT group, *P* = 0.590). The PV reconnection rate (57.8% vs. 68.0%; *P* = 0.175) was not significantly between the small and large EAT groups. The POBI reconnection was more frequent in the large EAT group (77.4%) than in the small EAT group (56.7%) (*P* = 0.034).

**TABLE 4:** Mapping findings in the patients undergoing repeat ablation procedures.

Redo mapping	Overall	Small EAT (≤116.23 mL)	Large EAT (>116.23 mL)	*P*-value
	(*n* = 205)	(*n* = 102)	(*n* = 103)	
Median duration between the *de novo* and redo procedures, months	20 (12–44)	22 (12–46)	20 (12–40)	0.589
PV reconnection, *n* (%)	129 (62.9)	59 (57.8)	70 (68.0)	0.175
1∼2 PV reconnections, *n* (% in PV reconnection)	95 (73.6)	43 (72.9)	52 (74.3)	1.000
3∼4 PV reconnections, *n* (% in PV reconnection)	34 (26.4)	16 (27.1)	18 (25.7)	
CTI reconnection, *n* (% in whom a CTI ablation was performed during the *de novo* procedure)	51/185 (27.6)	25/93 (26.9)	26/92 (28.3)	0.964
POBI reconnection, *n* (% in whom a POBI was performed during the *de novo* procedure)	75/113 (66.4)	34/60 (56.7)	41/53 (77.4)	0.034

CTI, cavotricuspid isthmus; EAT, epicardial adipose tissue; POBI, posterior wall box isolation; PV, pulmonary vein.

## Discussion

### Main findings

In this study, the beneficial association between an additional POBI in conjunction with a CPVI and lower recurrence risk exhibited a linear decrease with an increasing EAT volume (especially atrial EAT volume) in the patients with PeAF. Among the patients with a smaller EAT volume, the additional POBI was related to a 26% decreased risk for AF recurrence as compared to that with a CPVI alone. In contrast, among those with a greater EAT volume, the risk for AF recurrent did not differ between an additional POBI and a CPVI alone.

### Epicardial adipose tissue and atrial fibrillation

An EAT accumulation has been associated with the prevalence, severity, and recurrence of AF (3, 4, 7, 8). Basic and translational studies have suggested infiltration of adipocyte, oxidative stress, paracrine effects, autonomic dysfunction, and other pathways as arrhythmogenic mechanisms (13, 14). EAT acts as sources of inflammatory mediators and the adiposity-induced inflammation has been regarded to responsible for AF (15). Nalliah et al. reported that local myocardial infiltration of the EAT is related to conduction slowing and heterogeneity (16). Increased atrial EAT, especially, was associated with endothelial dysfunction in patients with AF (17). Kim et al. found that a high EAT volume in patients with PeAF predicted a poor clinical outcome after catheter ablation, but not in those with paroxysmal AF (PAF) (8). The role of the metabolic atrial substrate in the pathophysiology of PAF is less obvious than in PeAF. Different ablation approaches might be needed according to the amount and position of EAT, especially in PeAF.

### Role of additional linear ablation

Previous RCTs that investigated whether an empirical POBI improved the rhythm outcomes for PeAF or PeAF converted to PAF have demonstrated no improvements following the addition of a POBI, suggesting that a uniform empirical linear ablation is not beneficial (11, 18). Therefore, identifying patients who may benefit from additional linear ablation remains an important clinical question. Since the suggested mechanism elucidating the association between the EAT and AF is mainly that EAT contributes to a remodeled atrial substrate (14), the lower efficacy of a POBI (i.e., substrate modification) among patients with a large EAT observed in this study appears to be a paradox. In the study of Nakatani et al., EAT overlap on POBI lines is related to a high AF freedom rate after performing POBI and CPVI (19). However, there was no comparator group receiving CPVI alone in the study. Thus, the additional benefits from POBI according to varying EAT volume were not assessed. In contrast, the findings of this study suggested that substrate modification can be effective when performed in patients with a relatively lesser burden of atrial remodeling.

### Epicardial adipose tissue and reconnections of posterior wall box isolation

There have been several issues related to the substantially high reconnection rates after linear ablation. Pambrun et al. reported that conduction gaps through the roof line are common (33%) and are related to preserved epicardial conduction *via* the septopulmonary bundle (20). The low electrical and thermal conductivity properties characteristic of fat could make it difficult for radiofrequency current and heat conduction to penetrate to underlying tissue. An *in vitro* study by Suarez et al. demonstrated that increasing EAT was associated with a decrease of the tissue temperature and the depth of the lesion in the atrial wall induced by radiofrequency current at constant voltage (21). In line with these reports, Oudin et al. observed that the ablation procedure tended to be longer when EAT is more abundant (22). Our previous studies have reported a reconnection rate of up to 50% with a POBI during redo mapping (23). In this study, the reconnection rate of a POBI was 66.4% among those who underwent repeat ablation procedures. When stratified into three groups according to total EAT volume, a larger EAT was associated with a greater POBI reconnection rate (P for trend = 0.022) with reconnection rates of 84.2 and 59.5% in the largest and smallest EAT groups, respectively (Supplementary Table 3). Among the patients with a large EAT volume in this study, the early recurrence rate was significantly lower in the additional POBI group than in the CPVI alone group, despite no difference in the risk for clinical recurrence between the groups. This finding also suggested the possibility of frequent POBI reconnections during the follow-up in patients with a large EAT volume. Such high reconnection rates might explain the lower efficacy of an additional POBI in patients with a greater EAT volume. A future study investigating the association between the EAT and long-lasting permanent POBI is warranted.

### Study limitations

This study had several limitations. First, this was a single-center retrospective cohort study; consequently, the findings cannot be used to establish causal relationships and might have involved selection bias. Second, the proportion of patients with a BMI over 30 kg/m^2^ was smaller in this study (7.8%) than observed in Western countries. Third, the exact cumulative AF burden could not be assessed in this study. Fourth, even after propensity score weighting, residual confounding may persist. Unmeasured confounders might have influenced the findings (e.g., body weight changes during follow-up).

## Conclusion

In patients with PeAF, an additional POBI was associated with less frequent AF recurrences than a CPVI alone among the patients with smaller total EAT volumes. The beneficial association between an additional POBI and the rhythm outcomes exhibited a linear decrease with an increasing EAT volume. These results suggested that different ablation approaches could be considered in relation to the amount of epicardial fat.

## Data availability statement

The data analyzed in this study is subject to the following licenses/restrictions: Data, analytic methods and study materials are available upon reasonable request to other researchers who want to reproduce the results or replicate the procedure. Requests to access these datasets should be directed to the corresponding author.

## Ethics statement

The studies involving human participants were reviewed and approved by the study protocol adhered to the Declaration of Helsinki and was approved by the Yonsei University Health System’s institutional review board. The patients/participants provided their written informed consent to participate in this study.

## Author contributions

DK and H-NP contributed to the conception and design of the work, interpretation of data, and drafting of the manuscript. O-SK contributed to the acquisition and analysis of data. HY, T-HK, J-SU, BJ, and M-HL contributed to the conception and design of the work and revision of the manuscript. All authors contributed to the article and approved the submitted version.

## Footnotes

1
www.R-project.org
