# Corrigendum: Effect of epicardial fat volume on outcomes after left atrial posterior wall isolation in addition to pulmonary vein isolation in patients with persistent atrial fibrillation

**DOI:** 10.3389/fcvm.2023.1220717

**Published:** 2023-06-02

**Authors:** Daehoon Kim, Hee Tae Yu, Oh-Seok Kwon, Tae-Hoon Kim, Jae-Sun Uhm, Boyoung Joung, Moon-Hyoung Lee, Hui-Nam Pak

**Affiliations:** Division of Cardiology, Department of Internal Medicine, Yonsei University Health System, Seoul, Republic of Korea

**Keywords:** atrial fibrillation, epicardial adipose tissue, posterior wall 1solation, catheter ablation-atrial fibrillation, rhythm outcome

A Corrigendum on Effect of epicardial fat volume on outcomes after left atrial posterior wall isolation in addition to pulmonary vein isolation in patients with persistent atrial fibrillation by Kim D, Yu HT, Kwon OS, Kim TH, Uhm JS, Joung B, Lee MH, Pak HN. (2022) Front. Cardiovasc. Med. 9:1005760. doi: 10.3389/fcvm.2022.1005760

In the published article, there was an error. Incorrect numbers were used.

A correction has been made to **Results**, *Additional posterior wall box isolation in patients with a larger epicardial adipose tissue volume*, Paragraph 2. This sentence previously stated:

“After overlap weighting, the early recurrence was less frequent in the additional POBI group (38.6%) than in the CPVI alone group (52.4%) (*P* = 0.001) (Table 3).”

The corrected sentence appears below:

“After overlap weighting, the early recurrence was less frequent in the additional POBI group (39.4%) than in the CPVI alone group (54.1%) (*P* = 0.001) (Table 3).”

The authors apologize for this error and state that this does not change the scientific conclusions of the article in any way. The original article has been updated.

