# Supplementary material for: Effect of epicardial fat volume on outcomes after left atrial posterior wall isolation in addition to pulmonary vein isolation in patients with persistent atrial fibrillation
Source: Front Cardiovasc Med. 2022 Oct 26;9:1005760. doi: 10.3389/fcvm.2022.1005760 (PMC9643695; doi:10.3389/fcvm.2022.1005760)
Supplement: Supplementary file 1 [file Data_Sheet_1.docx]

**Supplementary Methods**

***Electrophysiological mapping and radiofrequency catheter ablation***

Intracardiac electrograms were recorded using the Prucka CardioLab Electrophysiology system (General Electric Medical Systems, Inc., Milwaukee, WI, USA). Three-dimensional electroanatomic mapping (NavX, St. Jude Medical, Inc., Minnetonka, Minnesota and CARTO, Biosense-Webster, Inc., Diamond Bar, California) was performed using a circumferential PV mapping catheter (Lasso, Biosense-Webster Inc.) through a long sheath (Schwartz left 1, St. Jude Medical, Inc.). Systemic anticoagulation was achieved with intravenous heparin to maintain an activated clotting time of 350–400 s during the procedure. An open-irrigated tip catheter (Celsius [Johnson & Johnson, Inc., Diamond Bar, CA]; NaviStar ThermoCool [Biosense Webster, Inc]; ThermoCool SF [Biosense Webster, Inc]; Coolflex [St. Jude Medical, Inc]; FlexAbility [St. Jude Medical, Inc]; ThermoCool SmartTouch [Biosense Webster, Inc.]; and TactiCath [St. Jude Medical, Inc]) was used. Since the study patients were observed for a relatively long period, the RF power for the AFCA varied between 25 W and 60 W.

***Post-ablation management, follow-up, and clinical outcome***

We discharged the patients without receiving AADs except for those who had recurrent extra-PV triggers after the AFCA procedure, symptomatic frequent atrial premature beats, non-sustained atrial tachycardia, or an early recurrence of AF on telemetry during the admission period. An ECG was performed in all patients visiting the outpatient clinic at 1, 3, 6, and 12 months following the AFCA and every 6 months thereafter or whenever symptoms developed. Twenty-four-hour Holter recordings were performed at 3, 6, and 12 months and every 6 months thereafter. Patients who reported episodes of palpitations suggestive of an arrhythmia recurrence received Holter monitoring or event monitoring recordings.

***Propensity overlap weighting***

The propensity score, which is the probability of receiving an additional POBI, was estimated using a logistic regression based on the age, sex, BMI, CHA_2_DS_2_-VASc, medical history, echocardiographic parameters, and total EAT volume. Continuous variables were modelled as cubic spline functions. The distribution of the propensity scores before and after overlap weighting is shown in Supplemental Figure 1. The overlap weight was calculated as 1 minus the propensity score for patients in the additional POBI group or as the propensity score for patients in the CPVI only group to obtain the estimates representing the population average of the treatment effects with a minimized asymptotic variance of the treatment effect and a desirable exact balance property. The balance between the treatment populations was evaluated by the standardized differences of all baseline covariates using a threshold of 0.1 to indicate an imbalance.

**Supplementary Table 1.** Baseline characteristics of patients with small epicardial adipose tissue before and after propensity overlap weighting.

| **Variables** | **Small EAT (≤116.23mL) (n=594)** | | | | | | | |
| --- | --- | --- | --- | --- | --- | --- | --- | --- |
|  | **Crude** | | | | **After overlap weighting** | | | |
|  | **CPVI only** | **Additional POBI** | **ASD** | **P value** | **CPVI only** | **Additional POBI** | **ASD** | **P value** |
|  | **(n=319)** | **(n=275)** |  |  | **(n=319)** | **(n=275)** |  |  |
| Age, years | 59 (52–66) | 59 (52.50–66) | 2.8% | 0.993 | 59 (52–65) | 58 (52–65) | <0.1% | 0.779 |
| Female | 81 (25.4) | 75 (27.3) | 4.3% | 0.670 | 25.9 | 25.9 | <0.1% | 1.000 |
| BMI, kg/m^2^ | 24.2 (22.5–26.0) | 24.0 (22.6–25.8) | 5.0% | 0.785 | 24.2 (22.5–25.9) | 24.1 (22.6–25.8) | <0.1% | 0.859 |
| CHA_2_DS_2_-VASc score | 2 (1–2) | 1 (1–3) | 6.5% | 0.644 | 2 (0–2) | 1 (1–2) | 1.6% | 0.970 |
| Comorbidities, |  |  |  |  |  |  |  |  |
| Heart failure | 68 (21.3) | 54 (19.6) | 4.2% | 0.687 | 19.6 | 19.6 | <0.1% | 1.000 |
| Hypertension | 137 (42.9) | 119 (43.3) | 0.7% | 1.000 | 42.7 | 42.7 | <0.1% | 1.000 |
| Diabetes mellitus | 47 (14.7) | 36 (13.1) | 4.7% | 0.648 | 13.7 | 13.7 | <0.1% | 1.000 |
| Stroke | 33 (10.3) | 30 (10.9) | 1.8% | 0.929 | 10.2 | 10.2 | <0.1% | 1.000 |
| Vascular disease | 18 (5.6) | 31 (11.3) | 20.3% | 0.019 | 7.7 | 7.7 | <0.1% | 1.000 |
| Echocardiographic parameters |  |  |  |  |  |  |  |  |
| LA dimension, mm | 42 (38–46) | 44 (40–48) | 34.6% | <0.001 | 43 (39–47) | 44 (39–47) | <0.1% | 0.985 |
| LVEF, % | 62 (57–67) | 62 (58–67) | 0.5% | 0.801 | 62 (57–67) | 62 (57–66) | <0.1% | 0.961 |
| E/Em | 9.0 (7.2–10.8) | 9.2 (7.2–12.1) | 22.8% | 0.019 | 9 (7–12) | 9 (7–12) | <0.1% | 0.985 |
| Total EAT volume, mL | 89.27 (70.25–104.28) | 84.85 (68.25–100.35) | 16.4% | 0.031 | 86.61 (68.49–102.91) | 86.97 (70.37–100.87) | <0.1% | 0.840 |
| Atrial EAT volume, mL | 36.74 (29.63–43.92) | 34.30 (26.58–41.10) | 22.7% | 0.006 | 35.51 (27.32–42.48) | 35.54 (27.23–42.40) | <0.1% | 0.967 |
| Ventricular EAT volume, mL | 50.94 (40.72–59.49) | 50.12 (39.07–58.89) | 8.0% | 0.340 | 50.73 (39.26–59.49) | 50.72 (40.21–58.95) | <0.1% | 0.939 |

Values are presented as median (interquartile range), n (%), or %.

BMI = body mass index; CPVI = circumferential pulmonary vein isolation; EAT = epicardial adipose tissue; E/Em = mitral inflow velocity/mitral annulus tissue velocity; LA = left atrium; LVEF = left ventricle ejection fraction; POBI = posterior wall box isolation.

**Supplementary Table 2.** Baseline characteristics of patients with large epicardial adipose tissue before and after propensity overlap weighting.

| **Variables** | **Large EAT (>116.23mL) (n=593)** | | | | | | | |
| --- | --- | --- | --- | --- | --- | --- | --- | --- |
|  | **Crude** | | | | **After overlap weighting** | | | |
|  | **CPVI only** | **Additional POBI** | **ASD** | **P value** | **CPVI only** | **Additional POBI** | **ASD** | **P value** |
|  | **(n=368)** | **(n=225)** |  |  | **(n=368)** | **(n=225)** |  |  |
| Age, years | 60 (53–67.25) | 60 (53–68) | 0.1% | 0.890 | 60 (54–66) | 60 (53–68) | <0.1% | 0.868 |
| Female | 54 (14.7) | 32 (14.2) | 1.3% | 0.975 | 13.8 | 13.8 | <0.1% | 1.000 |
| BMI, kg/m^2^ | 26.5 (24.5–28.4) | 26.7 (24.8–28.5) | 7.2% | 0.533 | 26.7 (24.7–28.6) | 26.7 (24.8–28.4) | <0.1% | 0.819 |
| CHA_2_DS_2_-VASc score | 2 (1–3) | 2 (1–3) | 4.8% | 0.849 | 2 (1–3) | 2 (1–3) | 2.6% | 0.951 |
| Comorbidities, |  |  |  |  |  |  |  |  |
| Heart failure | 86 (23.4) | 41 (18.2) | 12.7% | 0.168 | 19.0 | 19.0 | <0.1% | 1.000 |
| Hypertension | 211 (57.3) | 143 (63.6) | 12.7% | 0.158 | 61.4 | 61.4 | <0.1% | 1.000 |
| Diabetes mellitus | 101 (27.4) | 46 (20.4) | 16.5% | 0.069 | 23.4 | 23.4 | <0.1% | 1.000 |
| Stroke | 33 (9.0) | 27 (12.0) | 9.9% | 0.295 | 10.7 | 10.7 | <0.1% | 1.000 |
| Vascular disease | 35 (9.5) | 35 (15.6) | 18.3% | 0.037 | 13.7 | 13.7 | <0.1% | 1.000 |
| Echocardiographic parameters |  |  |  |  |  |  |  |  |
| LA dimension, mm | 44 (41–48) | 48 (44–51) | 56.0% | <0.001 | 46 (43–49) | 46 (42–50) | <0.1% | 0.821 |
| LVEF, % | 62 (58–65.62) | 62 (57–67) | 2.0% | 0.962 | 62 (58–65) | 62 (57–67) | <0.1% | 0.679 |
| E/Em | 9.53 (8–12.17) | 10 (8–13.29) | 13.8% | 0.038 | 10 (8–12) | 10 (8–13) | <0.1% | 0.634 |
| Total EAT volume, mL | 150.1 (132.5–170.7) | 148.4 (132.5–178.8) | 6.7% | 0.752 | 150.3 (132.4–171.4) | 146.3 (129.2–176.3) | <0.1% | 0.575 |
| Atrial EAT volume, mL | 63.6 (55.0–73.6) | 61.9 (52.4–75.2) | 1.2% | 0.190 | 62.5 (54.4–73.1) | 61.5 (52.3–73.4) | <0.1% | 0.444 |
| Ventricular EAT volume, mL | 85.4 (75.1–100.6) | 88.0 (75.2–105.7) | 11.6% | 0.438 | 85.8 (75.6–101.8) | 86.3 (73.7–103.9) | <0.1% | 0.894 |

Values are presented as median (interquartile range), n (%), or %.

BMI = body mass index; CPVI = circumferential pulmonary vein isolation; EAT = epicardial adipose tissue; E/Em = mitral inflow velocity/mitral annulus tissue velocity; LA = left atrium; LVEF = left ventricle ejection fraction; POBI = posterior wall box isolation.

**Supplementary Table 3. Mapping findings of patients undergoing repeat ablation procedures stratified into three groups according to the volume of epicardial adipose tissue measured at de novo procedures.**

| **Redo mapping** | **EAT 1st tertile** | **EAT 2nd tertile** | **EAT 3rd tertile** | **P value*** | **P for trend**† |
| --- | --- | --- | --- | --- | --- |
|  | **(n=64)** | **(n=63)** | **(n=78)** |  |  |
| Duration between the de novo and redo, months | 23 (12–50) | 20 (11–37) | 19 (12–44) | 0.486 | 0.454 |
| PV reconnection, n (%) | 37 (57.8) | 40 (63.5) | 52 (66.7) | 0.551 | 0.281 |
| 1~2 PV reconnections, n (% in PV reconnection) | 28 (75.7) | 27 (67.5) | 40 (76.9) | 0.564 | 0.817 |
| 3~4 PV reconnections, n (% in PV reconnection) | 9 (24.3) | 13 (32.5) | 12 (23.1) |  |  |
| CTI reconnection, n (% in whom CTI was performed at de novo procedures) | 15/59 (25.4) | 15/58 (25.9) | 21/68 (30.9) | 0.743 | 0.484 |
| POBI reconnection, n (% in whom POBI was performed at de novo procedures) | 25/42 (59.5) | 18/33 (54.5) | 32/38 (84.2) | 0.015 | 0.022 |

BDB, bidirectional block; CTI, cavotricuspid isthmus; EAT, epicardial adipose tissue; POBI, posterior box isolation; PV, pulmonary vein.

*Estimated using Kruskal-Wallis Rank Sum test or Fisher’s exact test.

†Estimated using Spearman correlation or Cochran-Armitage trend test.

**Supplementary Figure 1.** An example of EAT measurements of separated atriums and ventricles.

**
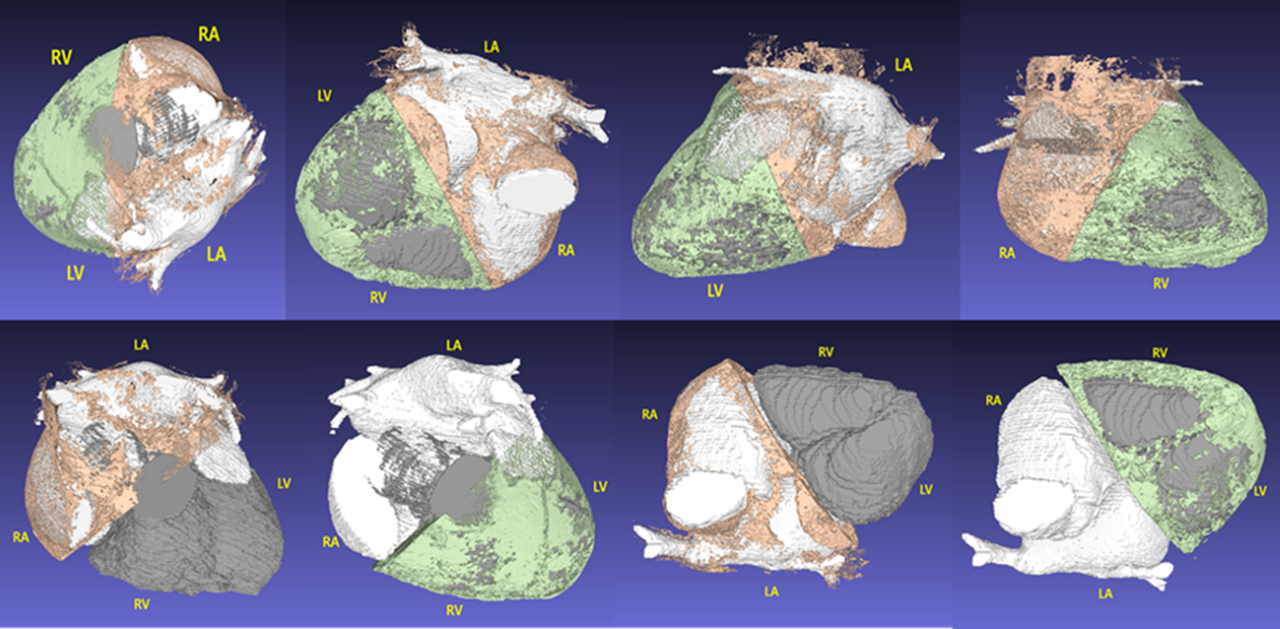
**

**Supplementary Figure 2.** Distributions of the propensity scores before and after overlap weighting in (A) patients with small epicardial fat volume and (B) with large epicardial fat volume.

**(A) Small epicardial fat volume (≤116.23mL)**

**
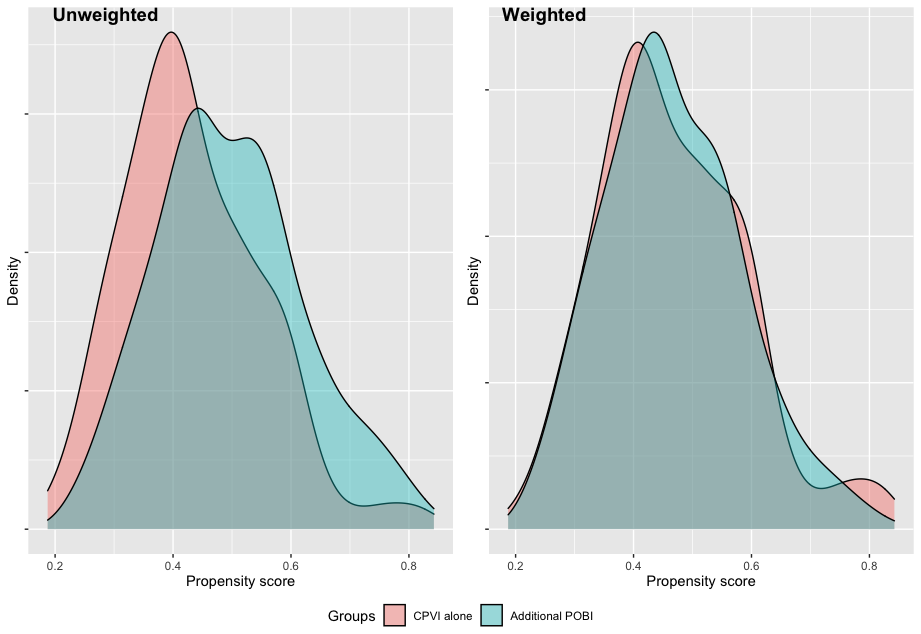
**

**(B) Large epicardial fat volume (>116.23mL)**

**
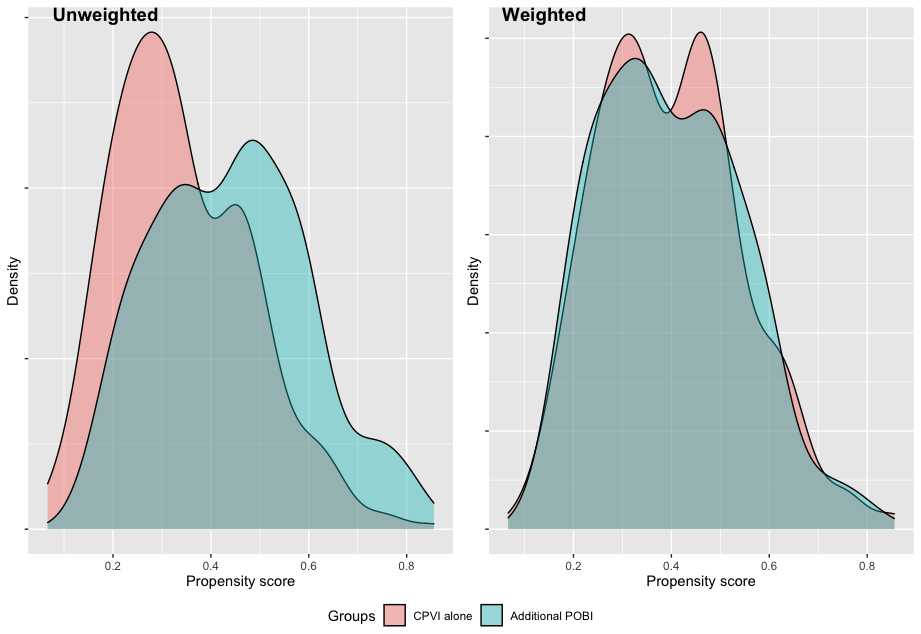
**
